# Dopamine-Mediated Sclerotization of Regenerated Chitin in Ionic Liquid

**DOI:** 10.3390/ma6093826

**Published:** 2013-09-06

**Authors:** Dongyeop X. Oh, Sara Shin, Chanoong Lim, Dong Soo Hwang

**Affiliations:** 1 POSTECH Ocean Science and technology Institute, Pohang University of Science and Technology, Pohang 790-784, Korea; E-Mail: fantasista@postech.ac.kr; 2 School of Environmental Science and Engineering, Pohang University of Science and Technology, Pohang 790-784, Korea; E-Mail: sarashin@postech.ac.kr; 3 School of Interdisciplinary Bioscience and Bioengineering, Pohang University of Science and Technology, Pohang 790-784, Korea; E-Mail: marine983@postech.ac.kr

**Keywords:** dopamine, regenerated chitin, sclerotization, water-absorption, mechanical properties, ionic liquid

## Abstract

Chitin is a promising structural material for biomedical applications, due to its many advantageous properties and abundance in nature. However, its usage and development in the biomedical field have been stagnant, because of chitin’s poor mechanical properties in wet conditions and the difficulties in transforming it into an applicable form. To overcome these challenges, we created a novel biomimetic chitin composite. This regenerated chitin, prepared with ionic liquid, showed improved mechanical properties in wet conditions by mimicking insect cuticle and squid beak sclerotization, *i.e.*, catechol-meditated cross-linking. By ionic liquid-based heat treatment, dopamine oxidation produced melanin-like compounds and dopamine-meditated cross-links without any solvent evaporation and oxidant utilization. The dopamine-meditated sclerotization increased the ultimate tensile strength (UTS) of the regenerated chitin by 2.52-fold, measured after six weeks of phosphate-buffered saline (PBS) submersion. In addition, the linear swelling ratio (LSR) of the chitin film was reduced by about 22%. This strategy raises a possibility of using regenerated chitin as an artificial hard tissue in wet conditions.

## 1. Introduction

Generally, chitin (a linear structural polysaccharide mainly composed of poly-*n*-acetyl-d-glucosamine connected by β-1,4 linkages) is one of the main hard-tissue components of crustaceans, insects and fungi [1]. Due to its superior mechanical properties (~50 GPa of stiffness) [2], abundance, biocompatibility, biodegradability, wound-healing activity and antibacterial activity, chitin is a promising structural material for biomedical application [3]. 

However, chitin processing methods are limited, because it is neither meltable nor soluble in most solvents. Only solvents, such as NaOH/urea (aq) and ionic liquids, have been found to successfully dissolve chitin [4,5,6,7,8,9]. Among them, ionic liquid has been considered the best solvent for chitin. Ionic liquid can hold a higher concentration (~40 wt %) of chitin compared to the other solvents, which enhances the process efficiency. Furthermore, ionic liquid is an eco-friendly solvent, as it is recyclable and generates hardly any air-pollutants, due to its non-volatility, low-reactivity and low-flammability. In addition, this process has been shown to directly convert raw crustacean waste from crab and shrimp into chitin products without the need for purification [10].

The regenerated chitin prepared with ionic liquid, however, acquires poor mechanical properties and a high extent of swelling in wet conditions [5]. Thus, chitin has not been successfully commercialized as a biomedical material. Indeed, when the regenerated chitin was tested as an artificial tendon or ligament *in vivo*, it softened and eventually broke apart in the physiological environment of blood and lymph within two months [11]. 

The recently discovered chemistry of natural chitin composites provides a new inspiration to improve the mechanical properties of regenerated chitin in wet conditions. Through the biochemical process called “sclerotization”, wet insect cuticle achieves a Young’s modulus greater than most commercial plastics at 20 GPa and a Vicker’s hardness comparable to human dentin at 0.25–0.78 GPa [12]. Insect cuticle is composed of chitin filaments, proteins and phenolic tanning compounds [13]. During the sclerotization of insect cuticle, oxidation of catechol moieties improves mechanical properties in wet conditions by producing hydrophobic polyphenolic compounds, such as melanin, and cross-linking reactions with chitin filaments and proteins [14,15]. Similarly, high stiffness of about five to 10 GPa was obtained even in the fully hydrated beak of the squid, *Dosidicus*, as a result of high-density catecholic-histidine cross-linking and dehydration in the matrix [16,17]. 

In this study, we report a chitin composite that can be used as biomedical implants, specifically, as artificial ligament or tendon. Mechanical properties and resistance to swelling of regenerated chitin in wet conditions were improved by biomimetic sclerotization, *i.e.*, the oxidation of dopamine, a physiological catecholic material [18]. Dopamine-meditated cross-links and melanin-like compounds were produced in this composite via heat-induced dopamine oxidation in recyclable 1-ethyl-3-methylimidazolium acetate ([C2mim] [OAc], an ionic liquid) solution without using toxic cross-linking agents, such as aldehyde, glutaraldehyde and periodate [19,20]. 

## 2. Results and Discussion

### 2.1. Fabrication of Regenerated Chitin Films

**Figure A1:**
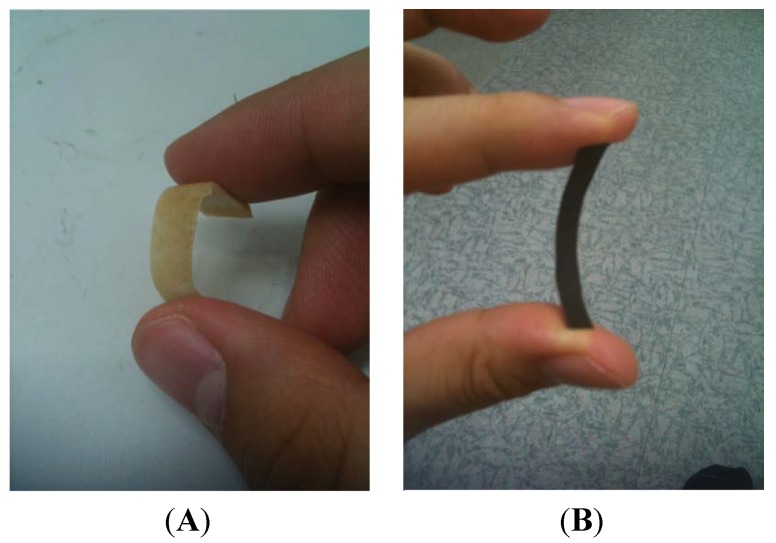
Pure regenerated chitin film (**A**) and 10 wt % dopamine-containing composite (**B**).

Regenerated films of pure chitin and dopamine-containing composites were successfully fabricated via the ionic liquid process. During heat treatment at 150 °C, the volume of chitin/ionic liquid solutions did not change, even without the use of a stopper on the reactors, indicating that the solvent is nonvolatile. This suggests that the nonvolatile property and high chitin-holding capacity of ionic liquid could reduce the generation of air-pollutants and improve processing efficiency [5,6,7,8,9]. The fully dried films had about 0.25 mm of thickness and were free-standing. The colors of pure chitin and dopamine-containing composite films were light-yellow and black, respectively (Figure A1). Previous reports suggest that the color-darkening may be related to the formation of phenolic tanning compounds, which could give rise to the hydrophobicity of biomaterials [15,21,22]. The derivatives from dopamine oxidation are widely known to couple with each other, producing phenolic compounds, such as the melanin-like compound [14,18,23]. 

### 2.2. Quantification of Melanin-Like Compound

To confirm formation of phenolic compounds, such as the melanin-like compound, a typical melanin quantification assay was conducted on 10 wt % dopamine-containing composites [24]. Acid hydrolysis treatment hydrolyzed everything, but the melanin-like compound. The remaining melanin was degraded by alkaline peroxidation to produce pyrrole acids, an orange-brown chromophore, in proportion to the melanin-like compound content. The melanin-like compound content was quantified by comparing its chromophore light absorption at 560 nm to that of *Sepia* melanin, which was used as the standard. The result showed that the chitin composite film contained about 9.1 wt % melanin-like compound, which implies that approximately 90% of the added dopamine in the film was converted. Catecholic oxidation to the melanin-like compound was successfully performed by heat treatment without using toxic oxidants, such as NaIO_4_ [25]. 

### 2.3. Spectral Evidences for Dopamine-Meditated Cross-Links and Melanin-Like Compound Formation

**Figure 1:**
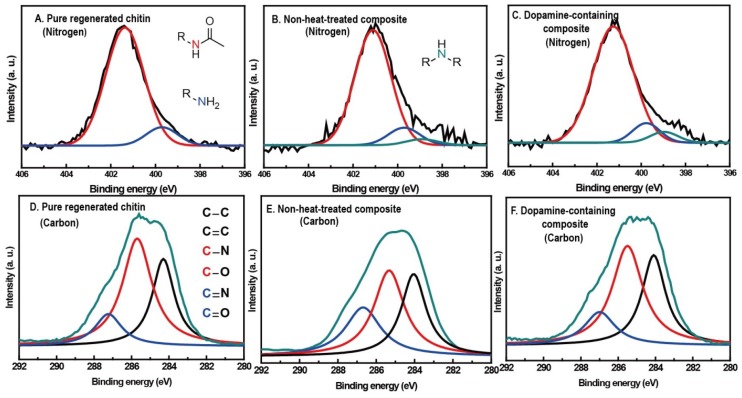
Nitrogen 1s narrow scans in X-ray photoelectron spectroscopy (XPS) spectra of (**A**) pure chitin; (**B**) non-heat-treated 10 wt % dopamine-containing composite; and (**C**) 10 wt % dopamine-containing composite. Carbon 1s narrow scans in XPS spectra of (**D**) pure chitin; (**E**) non-heat-treated 10 wt % dopamine-containing composite; and (**F**) 10 wt % dopamine-containing composite.

X-ray photoelectron spectroscopy (XPS) was used to examine the change of surface chemistry in chitin by dopamine addition and/or heat treatment (Figure 1A–C) [21,26]. Dopamine oxidative derivatives are widely known to couple with amine groups via Michael addition or Schiff base formation [27]. Therefore, N 1s narrow scans in XPS spectra of pure chitin, 10 wt % dopamine-containing composite and non-heat-treated 10 wt % dopamine-containing composite were obtained to monitor the amine group, a functional group of the deacetylated unit in chitin. Generally, the nitrogen spectrum could be classified into three peaks: 401.4 eV (red) for the nitrogen of acetyl amide group, 399.6 eV (blue) for the amine group and 398.6 eV (cyan) for R–N–C (or R–N=C). The ratio of the three peaks from pure chitin was about 100:10.9:0, implying that the degree of deacetylation of pure chitin was about 10%. Ten weight percent of dopamine-containing composite gave the ratio of 100:8.2:3.8, which indicated lower amine concentration and higher R–N–C (or R–N=C) concentration in comparison with the none-heat-treated composite’s ratio of 100:9.5:1.2. This suggests that heat-induced oxidation produces dopamine cyclization and coupling reactions between the amine group of chitin and the dopamine derivative, which could cross-link with the chitin matrix. To further confirm cross-links in the composite, solubility of pure chitin and 10 wt % dopamine-containing composite in DMAc/LiCl solution were compared. Ninety-two weight percent of pure chitin and 12 wt % of the composite were dissolved; this reduced solubility supports that dopamine derivatives cross-link with the chitin matrix.

**Figure A2:**
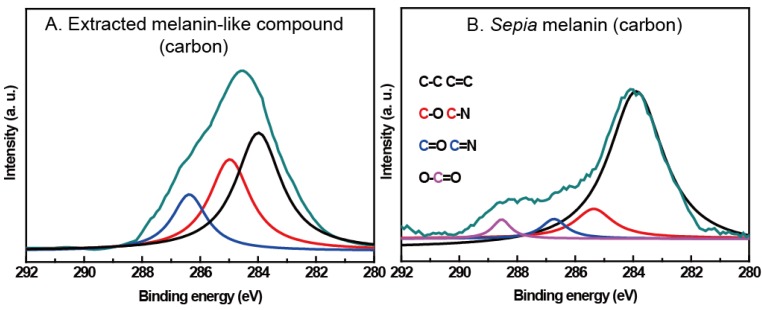
Carbon 1s narrow scans in XPS spectra of extracted melanin-like compound from 10 wt% dopamine containing composite (**A**) and *Sepia* melanin (control, **B**).

XPS narrow scan in the C s1 region of the same three samples with nitrogen examination were obtained by monitoring the aromatic group of dopamine and its derivatives. Similar to our previous XPS study on chitosan, pure chitin film showed three types of peak at 286.8 eV (A), 285.4 eV (B) and 284.3 eV (C) and a component ratio of 24.1:100:59.8, each assigned to the carbon atoms of the C=O or O–C–O group, the C–O or C–N group and R–C–R or C=C, respectively (Figure 1D–F). The component ratio of 10 wt % dopamine-containing composite was about 24.6:100:71.1, which is two-fold and 1.31-fold higher in (A) and (C) concentration than the concentration before heat treatment, respectively. The relative increase of (A) and (C) components, which indicate the C=O group and the aromatic group, implies that the heat treatment induced the transformation of dopamine to a melanin-like compound. XPS carbon narrow scan was also performed on *Sepia* melanin and the melanin-like compound. Both XPS spectra were comparable, indicating melanin-like compound formation in the composite (Figure A2). Overall, the XPS results reaffirm the melanin-like compound formation in the composite and support the cross-linking reaction between chitin and dopamine derivatives.

### 2.4. Contact Angle and Water Absorption

**Figure 2:**
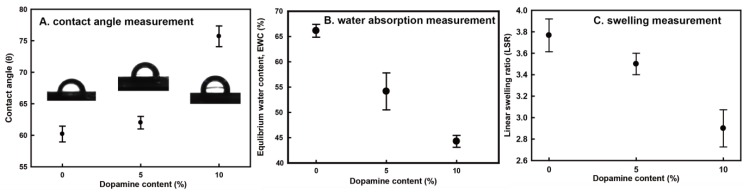
(**A**) Water contact angle; (**B**) equilibrium water content (EWC); and (**C**) linear swelling ratio (LSR) values of pure regenerated chitin, 5 wt % dopamine-containing composite and 10 wt % dopamine-containing composite. Each value represents the mean of five analyses and its standard deviation.

From a thermodynamic perspective, the hydrophobicity of a composite significantly affects water-absorption, which causes its poor mechanical properties and swelling behavior. To investigate the effects of both dopamine-meditated cross-links and melanin-like compounds on the hydrophobicity of chitin, the contact angle was measured on the surface of pure chitin and 10 wt % dopamine-containing composite films (Figure 2A). Generally, pure chitin has variable contact angle values of 30° to 70°, depending on the processing method [28,29,30]. Our regenerated chitin achieved about a 60.3° contact angle, and dopamine oxidation increased the contact angle up to about 75.7°. This result suggests that the hydrophilic amine groups of chitin were capped with oxidative dopamine derivatives and that the melanin-like compounds increased the degree of hydrophobicity in chitin. Along with the hydrophobicity examination of the samples, equilibrium water content (EWC) and linear swelling ratio (LSR) values of chitin and the composites were obtained in order to investigate the effects of cross-links and melanin-like compounds on the water adsorption of chitin composites (Figure 2B,C) [26,31,32]. The EWC value decreased from about 66 to 44 with increasing dopamine content, implying that the increased hydrophobicity and cross-linking retarded the water absorption of chitin, which reduces the mechanical properties. Resistance to swelling is important for biomedical applications (e.g., artificial tendon or ligament materials), because dimensional change, *i.e.*, swelling, brings about distortion and decreases the mechanical properties of the material. The degree of dimensional change of the samples was normalized by LSR. As expected from the EWC data, the LSR value of dopamine-containing composites decreased by 1.2-fold from that of pure chitin. This suggests that the decrease of water absorption reduces the swelling behavior.

In summary, dopamine-meditated cross-links and hydrophobic melanin compounds decreased water absorption in chitin, thereby improving the water swelling resistance of chitin film.

### 2.5. Crystal Structure and Morphology of Films

**Figure 3:**
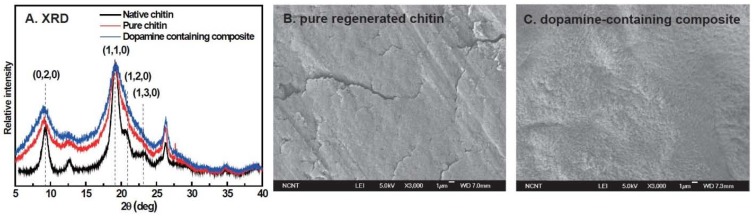
(**A**) X-ray diffraction (XRD) spectra of native chitin (black curve), pure regenerated chitin (red curve), 10 wt % dopamine-containing composite (blue curve); scanning electron microscope (SEM) images of (**B**) pure regenerated chitin; and (**C**) 10 wt % dopamine-containing composite.

To investigate the effects of the regeneration process and dopamine oxidation on chitin structure, wide-angle X-ray diffraction (WAXD) patterns of native chitin powder, pure chitin and 10 wt % dopamine-containing composite films were compared with each other (Figure 3A). The pattern of native chitin powder gave six evident peaks at around 9°, 12°, 18°, 21°, 26° and 35°, which are assigned to (020), (021), (110), (120), (130) and (152) planes, respectively [26]. This pattern is in agreement with the typical α-chitin crystal structure, which indicates that native chitin is mainly composed of α-chitin crystal. The WAXD pattern of pure chitin film expressed the observed six peaks, but they were weaker and broader than those of native chitin. This suggests that the regeneration of chitin transformed α-chitin crystal into a semi-α-chitin crystal and lowered the degree of crystallinity [26]. The WAXD pattern of dopamine-containing composite was comparable to that of chitin film, indicating that dopamine addition did not significantly affect the crystal structure of regenerated chitin since cross-linking reactions and melanin formation dominantly occur in the amorphous phase.

To further survey the effect of dopamine oxidation on chitin morphology, scanning electron microscope (SEM) images of the fractured surfaces of pure chitin and the 10% dopamine-containing composite were compared (Figure 3B). The surface morphology of pure chitin was relatively smooth and homogeneous except for some 200–300 μm-sized cracks. However, the surface of the dopamine-containing composite was relatively rough, probably due to the matrix shrinkage by dopamine-meditated cross-links.

### 2.6. Mechanical Properties of Chitin and Composites in Wet Conditions

**Figure 4:**
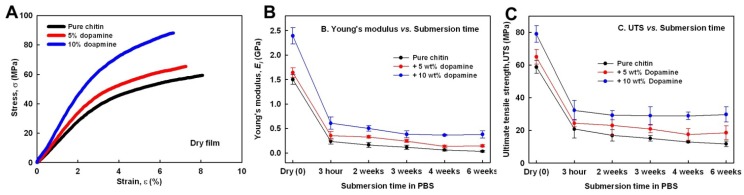
(**A**) Strain-stress curves of pure regenerated chitin (black curve), 5 wt % dopamine-containing composite (red curve) and 10 wt % dopamine-containing composite (blue curve); (**B**) Young’s modulus; and (**C**) ultimate tensile strength (UTS) values of pure regenerated chitin, 5 wt % dopamine-containing composite and 10 wt % dopamine-containing composite depending on submersion time in phosphate-buffered saline (PBS).

Although chitin has a good wound-healing property and bio-absorptivity, chitin-based material is not good enough to be an artificial hard tissue, due to its poor mechanical properties in wet conditions. Thus, we investigated whether or not the dopamine-meditated cross-links and the reduced water absorption will improve the mechanical properties of wet chitin. First, strain-stress curves of dry pure chitin and composites were obtained (Figure 4A). Young’s modulus and ultimate tensile strength (UTS) of dry chitin film were 1.5 ± 0.1 GPa and 59.42 ± 1.2 MPa, respectively, less than 3% of the mechanical properties of α-chitin crystal. This supports that chitin regeneration lowered the crystallinity and induced defective crystal structures, *i.e.*, semi-α-chitin [33]. The Young’s modulus and UTS each increased up to 2.4 ± 0.1 GPa and 79.04 ± 5.9 MPa with increasing dopamine content. This suggests that the cross-linking increased the mechanical properties of regenerated chitin and that the extent of the cross-links was in proportion to the dopamine content. Second, to determine the decrease in the mechanical properties of chitin and composites in physiologically wet conditions, tensile properties of chitin and composites at 37 °C depending on immersion time (3 h, two weeks, three weeks, four weeks and six weeks) in phosphate-buffered saline (PBS) were obtained. After 3 h, Young’s modulus and UTS of pure chitin were only 0.25 ± 0.05 GPa and 20.9 ± 5.1 MPa, respectively, about 34% of the dry chitin film level (Figure 4B). After six weeks, its Young’s modulus and UTS decreased to 0.08 ± 0.01 and 12 ± 1.5, respectively. Increasing dopamine content raised its Young’s modulus and UTS for each of the examined immersion times. After six weeks, inter alia, 10 wt % dopamine-containing composite achieved 0.37 ± 0.06 GPa of Young’s modulus and 29.4 ± 4.2 MPa of UTS, which were about three-fold higher than the level in PLA (poly-l-lactic acid), a representative synthetic biopolymer, in wet condition with 0.1 GPa of Young’s modulus and about 10 MPa of UTS. This suggests that dopamine-meditated cross-links and the reduced water-absorption improved tensile properties of fully wet chitin material.

### 2.7. Cytotoxicity Test on Dopamine-Containing Composite

**Figure 5:**
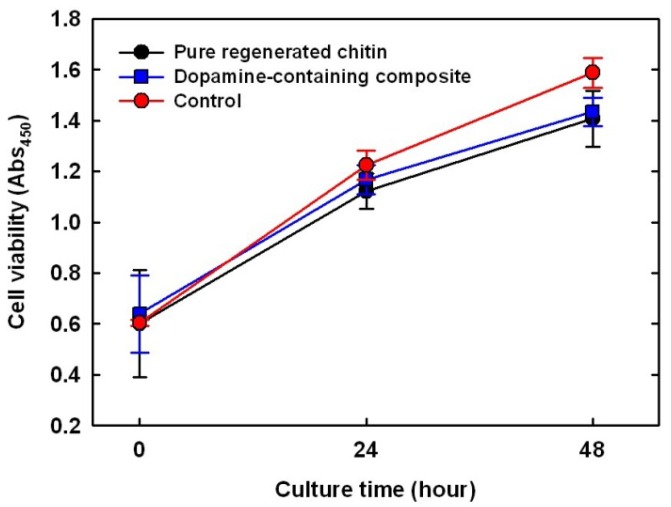
Comparison of MC3T3 cell proliferation on pure regenerated chitin (black); 10 wt % dopamine-containing composite (blue); and negative control (red, culture dish).

Glutaraldehyde and formaldehyde, widely used cross-linking agents for biomaterials, are limited in dosage due to their biological toxicity. Thus, to test the cytotoxicity of dopamine as a cross-linker in a mammalian cell line (MC3T3-E1), viable cells on an untreated cell culture well were used as control, while pure chitin and composites were monitored for 48 h via a colorimetric assay (Figure 5). The number of viable cells on the pure chitin film increased 2.7-fold over the 48 h, slightly lower than that of the empty well (*p* < 0.08), probably because the polystyrene surface (tissue culture well) absorbs serum proteins well [22]. As expected, there was no apparent difference in the number of viable cells with dopamine addition, indicating that dopamine and its oxidative derivatives showed no cytotoxicity to MC3T3-E1. The side effects of dopamine on the whole human body have been poorly documented, but at a rare occurrence, may cause irregular heartbeats and anxiety. Therefore, *in vivo* testing is required as a future experiment to prove the cytotoxicity of the dopamine containing composites.

### 2.8. Contribution of Dopamine Oxidation to Improve Mechanical Properties and Resistance to Swelling in Wet Conditions

Hydrophilic amine groups of chitin, easily hydrated in wet conditions, could reduce the mechanical properties of wet chitin. While the stiffness of fully dried chitin crystal ranges from 40 to 65 GPa, fully hydrated regenerated chitin achieved only about 10–20 MPa. Since this weakness has stagnated most applications of chitin as artificial hard tissue, we tried to develop a chitin-based composite with improved mechanical properties and resistance to swelling in water by mimicking the chemistry of dopamine oxidation in sclerotization of natural chitin composites—insect cuticle and squid beak. To minimize cytotoxicity, dopamine was selected as the catechol, and sclerotization was done in ionic liquid. Thermal-induced dopamine oxidation formed not only cross-linking between amine groups of chitin, but also hydrophobic melanin-like compounds in the composite. Thanks to dopamine-meditated cross-linking in the chitin film, Young’s modulus and UTS of chitin films increased about 1.6-fold and 1.3-fold, respectively. In cooperation with the cross-linking reactions, hydrophobic melanin decreased water-absorption and swelling, expressed in EWC and LSR. The reduced water-absorption improved not only the resistance to swelling, but also the mechanical properties of chitin film in wet conditions. Consequently, dopamine-containing composite after six weeks of submersion in PBS achieved up to 0.37 ± 0.06 GPa of Young’s modulus and 29.4 ± 4.2 MPa of UTS, which is about 3% of human bone strength and is comparable to human ligament strength [34,35]. This result holds out the possibility that dopamine-containing composite could be applied as artificial ligament material.

## 3. Experimental Section 

### 3.1. Material

Chitin from shrimp shells (degree of acetylation >90%), lithium chloride (LiCl), *Sepia* melanin, [C2mim][OAc], dimethylacetamide (DMAc) and dopamine were purchased from (Sigma-Aldrich, St. Louis, MO, USA). They were used as received without any purification, and deionized water (MiliQ, Bedford, OH, USA) was used to prepare aqueous solutions.

### 3.2. Regenerated Films of Pure Chitin and Composites

Two types of regenerated films were prepared: pure chitin and dopamine-containing chitin composite. A typical chitin film preparation using ionic liquid is as follows [33,36]. One gram of chitin powder and a desired amount of dopamine (0, 5, 10 wt % of chitin) were dissolved in 9 g of [C2mim][OAc] at 100 °C for 6 h with stirring. The solutions were poured into flat molds and then cooled down in ambient conditions for 1 day to form gels. To induce dopamine oxidation, the dopamine-containing solution mold was further heat-treated at 150 °C for 15 min before cooling down [25]. All gel-containing molds were soaked in ethanol for 6 h and, then, washed in distilled water. The wet films were dried at 50 °C in a convection oven overnight. As a control for XPS examination, non-heat-treated 10 wt % dopamine containing-composite was prepared; the heat-treatment at 150 °C for 15 min was omitted.

### 3.3. Quantification of Melanin-Like Compound and Solubility of Chitin and Composites in DMAc/LiCl Solvent

Melanin-like compound content in 10 wt % dopamine-containing chitin film was quantified by acid hydrolysis and alkaline peroxidation [24]. *Sepia* melanin, used as a standard, or dry composite was weighed, put into an ampule with 6 M HCl and 5% phenol as antioxidant, vacuum-sealed and hydrolyzed at 110 °C for two days. Insoluble residues after full hydrolysis were separated from the supernatant by centrifugation, washed with distilled water to remove the hydrolysate and, then, fully dried in a convection oven. The insoluble residues were suspended as 0, 0.1, 0.2, 0.5 and 1 mg/mL in alkaline peroxide cocktail (1 volume 10 N NaOH, 2 volume 30% H_2_O_2_ and 37 volume deionized water), incubated overnight at 70 °C and centrifuged at 14,000 rpm to remove residual solids. Light absorbance at 560 nm of the supernatant was measured. Standard curves were constructed using 0, 0.1, 0.2, 0.5 and 1 mg/mL of hydrolyzed *Sepia* melanin.

The solubility of pure chitin and 10 wt% dopamine-containing composite in DMAc/LiCl solution was measured as follows. LiCl was dissolved in DMAc to make 5 wt % at 120 °C for 24 h. Zero-point-five weight percent of each sample of the solvent was immersed in the prepared LiCl/DMAc solution at room temperature for 24 h, and the solution was centrifuged at 14,000 rpm. The precipitates of both samples were weighed after centrifugation to calculate dissolved content.

### 3.4. Chemistry Characterization on Film Surface

Chemistry on cross-sectional surfaces of chitin, 10 wt % dopamine-containing composite and non-heat-treated 10 wt % dopamine-containing composite was examined by X-ray photoelectron spectroscopy (XPS) using a PHI 5800 ESCA System at 2 × 10^−6^ tor with a monochromatic Al Kα (1486.6 eV) anode (250 W, 10 kV, 27 mA). All binding energies were tuned to the main hydrocarbon peak, C 1s (284.6 eV). All XPS spectra were evaluated using CasaXPS. The atomic ratio of different nitrogen (or carbon) components in pure chitin and composites were calculated by the area ratio of their detail peaks.

### 3.5. Characterization of Mechanical Properties

The tensile properties of the films with varying immersion time in phosphate-buffered saline (PBS) at 37 °C were measured by the universal tensile tester (UTS, Instron, Norwood, UK) [26,31,33]. All films were dried for 12 h in a vacuum oven prior to immersion. The dried films were cut into 30 mm × 5 mm rectangles and, then, immersed in PBS (1X solution, Thermo Scientific, Waltham, UK) for the desired time (0, 3 h, 2 weeks, 3 weeks, 4 weeks and 6 weeks). The immersed films were taken out, clamped onto the grips with 10 mm distance and loaded with a constant strain rate of 5 mm/min till failure. To calculate the tensile stress (σ, MPa, load per unit cross-sectional area) on the films, the thickness of the films was determined using a micrometer before the test.

### 3.6. Characterization of Water Swelling and Hydrophobicity

To characterize the degree of water-absorption of the films, the equilibrium water content (EWC) was measured [31,26]. A dry chitin or composite film with a dimension of 30 mm × 5 mm was weighed to an accuracy of 10^−4^ g and immersed in PBS for 1 day, and the immersed film was weighed again after removing excess water. EWC was calculated using the following Equation:

(1) $$\text{EWC}=\frac{W_{\text{t}}-W_{0}}{W_{\text{t}}}\times 100$$

where *W*_0_ is the weight of the sample before immersion and *W*_t_ is the weight of the sample after immersion at time *t*, when the weight of the sample reaches the equilibrium state. Chitin in PBS normally reaches equilibrium after 1 day.

Expansion of chitin films by water-absorption was normalized by the linear swelling ratio (LSR) [32]. The length of the dry film was measured to an accuracy of 10^−2^ mm and immersed in PBS for 1 day, and the length of the immersed film was measured again after removing excess water. LSR was calculated using the following Equation:

(2) $$\text{LSR}=\frac{L_{\text{t}}}{L_{0}}$$

where *L*_0_ is the length before immersion and *L*_t_ is the length after immersion at time *t*, when the weight of the sample reaches equilibrium state. Chitin in water normally reaches dimensional equilibrium after 6 h.

The static water contact angle was measured with a Phoenix 300 goniometer (Surface Electro Optics, Korea), using the sessile drop method to normalize the degree of hydrophobicity.

### 3.7. Characterization of Morphology and Crystal Structure

The morphology of the fractured surfaces of the films was studied by a high-resolution scanning electron microscope (SEM, JEOL JSM-7401F). The crystal structure of chitin and the composites was studied via the wide-angle X-ray diffraction (WAXD) experiment [33]. The experiment was conducted on an X-ray diffractometer (XRD) (D/MAX-2500/PC, Rigaku, Japan), in which a 40 kV/100 mA Ni-filtered Cu *K*_α_ radiation was employed. The WAXD patterns were recorded in the region of a scattering angle of 5° to 40° with a scanning speed of 4°/min.

### 3.8. *In Vitro* Cell Culture Study

The cell viability test on the surfaces of chitin and 10 wt % dopamine-containing composite was carried out [37]. The fully swollen film disks with the same diameter as the 24-well plate were immersed in ethanol for 1 h and washed in PBS just prior to cell seeding. A mouse pre-osteoblast cell line, MC3T3-E1, was cultured in minimal essential medium-alpha (MEM-α; Hyclone) supplemented with 10% fetal bovine serum (FBS; Hyclone) and 1% penicillin/streptomycin (Hyclone) at 37 °C under a humidified atmosphere of 5% CO_2_ and 95% air. The subconfluent cells were detached by 0.25% trypsin-EDTA (Hyclone), and the viable cells were counted by trypan blue assay. The cells were further seeded onto the film-containing wells and the empty wells for control in a 24-well plate at a density of about 3 × 10^4^ cells per well and cultured for 3 days. The number of viable cells depending on the culture time (0 to 3 days) was determined via a colorimetric assay (CCK-8, Dojindo); the number of viable cells is proportional to the light absorbance value at 450 nm.

## 4. Conclusions 

Here, we have investigated the effect of dopamine oxidation on regenerated chitin films by mimicking nature’s chitin composites. Dopamine oxidation of chitin films produced hydrophobic melanin-like compounds and cross-linking between chitin molecules, thereby improving the mechanical properties and resistance to swelling of regenerated chitin films in wet conditions. Notably, cross-links and melanin-like compound formation enhanced the Young’s modulus and UTS of regenerated chitin in wet conditions to a level compatible to human ligament without cytotoxicity. The heat-induced oxidation in ionic liquid is eco-friendly and economical, because neither of the processes demand oxidant addition nor provide solvent evaporation. This strategy expands the possible applications of the regenerated chitin as artificial hard tissues.
